# Living in a Foster Home: The Single Subpolar Flagellum Fla1 of *Rhodobacter sphaeroides*

**DOI:** 10.3390/biom10050774

**Published:** 2020-05-16

**Authors:** Laura Camarena, Georges Dreyfus

**Affiliations:** 1 Depto. Biología Molecular y Biotecnología, Instituto de Investigaciones Biomédicas, Universidad Nacional Autónoma de México, Ciudad Universitaria, CDMX 04510, Mexico; 2 Depto. Genética Molecular, Instituto de Fisiología Celular, Universidad Nacional Autónoma de México, Ciudad Universitaria, CDMX 04510, Mexico

**Keywords:** bacterial flagellum, *Rhodobacter sphaeroides*, motility, FliL, FlgT, flagellar rod, flagellar hook, FlgP

## Abstract

*Rhodobacter sphaeroides* is an α-proteobacterium that has the particularity of having two functional flagellar systems used for swimming. Under the growth conditions commonly used in the laboratory, a single subpolar flagellum that traverses the cell membrane, is assembled on the surface. This flagellum has been named Fla1. Phylogenetic analyses have suggested that this flagellar genetic system was acquired from an ancient γ-proteobacterium. It has been shown that this flagellum has components homologous to those present in other γ-proteobacteria such as the H-ring characteristic of the *Vibrio* species. Other features of this flagellum such as a straight hook, and a prominent HAP region have been studied and the molecular basis underlying these features has been revealed. It has also been shown that FliL, and the protein MotF, mainly found in several species of the family *Rhodobacteraceae*, contribute to remodel the amphipathic region of MotB, known as the plug, in order to allow flagellar rotation. In the absence of the plug region of MotB, FliL and MotF are dispensable. In this review we have covered the most relevant aspects of the Fla1 flagellum of this remarkable photosynthetic bacterium.

## 1. Introduction

### 1.1. The Flagellar Structure

**Figure 1:**
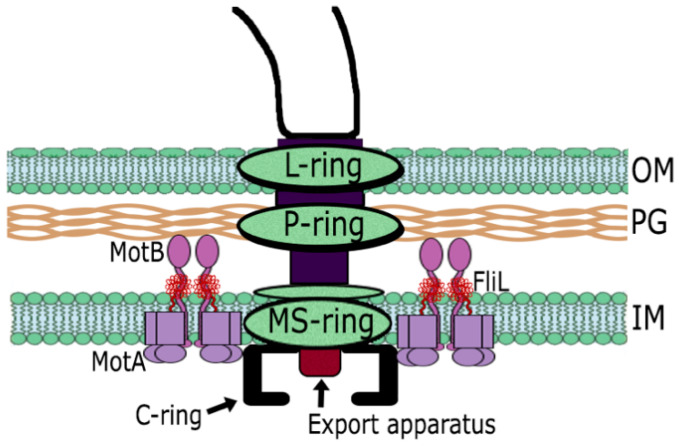
Scheme of the bacterial flagellum that shows the most relevant elements of the core structure that is common to several species of Gram-negative microorganisms.

The bacterial flagellum is driven by a complex molecular motor. The flagellar basal body contains the rotor and the export apparatus, and is composed of numerous proteins arranged in several rings and a central rod (reviewed recently in [1]). The MS ring, embedded in the internal membrane, is the base platform for the assembly of the rod. At the center of the MS ring a flagellar-specific export system is responsible for the export of most of the axial proteins that form the basal body [2,3,4,5]. The flagellar rod traverses the cell envelope and in its proximal end is formed by FlgB, FlgC and FlgF, and the distal end by FlgG [6]. Around the distal rod, the P and L rings act as a bushing allowing rod penetration through the peptidoglycan and the outer membrane, respectively [7,8]. This process is favored by the action of the bifunctional protein FlgJ that acts as a scaffolding rod-capping protein and also possesses glucosaminidase activity to penetrate the cell wall [9,10,11,12]. Once the rod reaches the outer membrane, the hook is assembled outside the cell. This structure transmits torque to the flagellar filament [13]. The physical properties of these two axial structures are different given that the filament is a long rigid helix and the hook is a short flexible structure that acts as a universal joint [14,15,16]. The distal end of the hook is connected to the filament via the hook associated proteins, FlgK and FlgL that mediate the transition from the flexible hook to the rigid filament [6,17]. The filament is the most prominent component, typically 5–10 μm in length and is several times longer than the cell body. The flagellar motor contains a stator that is composed by multiple units of the MotA/MotB complexes (4:2 stoichiometry) that form an ion channel that conducts the ions (H^+^ or Na^+^) of the transmembrane electrochemical gradient and generates motor rotation that propels the bacterial cell [1,18,19,20] (Figure 1). Recruitment of the MotA/B complexes and activation of the proton channel are complex processes that have been extensively reviewed recently [1,18,19,21]. Briefly, it is important to mention that recruitment of these complexes to the basal body has been related to the interaction of the cytoplasmic loop of MotA with FliG, which is part of the C-ring (Figure 1) [22,23,24]. Besides, in *Vibrio* the proteins MotY and MotX form the periplasmic T-ring that interacts with PomB (equivalent to MotB in *Vibrio*) and stabilize the stator complexes [25,26]. Activation of the proton channel requires extensive remodeling of the periplasmic region of MotB [27,28,29,30,31,32], and it has been proposed that the flagellar protein FliL participates in this process [33,34,35,36,37].

The flagellum has been thoroughly studied in various bacterial species, and recently the advancement of cryo-electron tomography, a powerful non-invasive technique, has revealed a high complexity and variability of its ultrastructural components [38,39,40,41,42,43,44].

### 1.2. The Two Flagellar Systems of Rhodobacter sphaeroides

**Figure 2:**
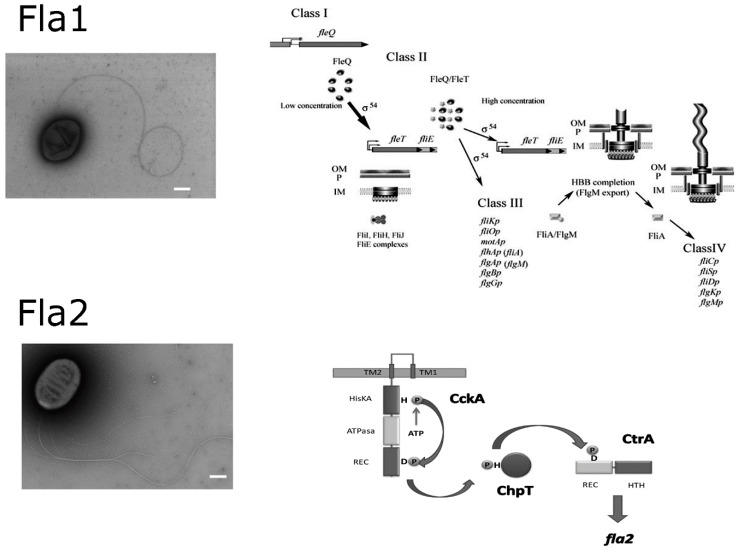
Electron micrograph showing *Rhodobacter sphaeroides* expressing either the Fla1 flagellum or Fla2 flagella. Cells were grown separately and under different growth conditions (for details see, [62]). Bar = 500 nm. The schemes showing the regulatory pathway of each flagellar system are shown at the right side of each micrograph [60,61].

*R. sphaeroides* is an α-proteobacterium from the non-taxonomic group of the purple non-sulfur photosynthetic bacteria. This microorganism frequently found in lakes and stagnant water bodies has a versatile metabolism since it grows by aerobic or anaerobic respiration, photosynthesis or fermentation [45]. The genome of several strains of *R*. *sphaeroides* has been sequenced and it consists of two chromosomes and several plasmids [46,47,48] or by one chromosome, one chromide and several plasmids as it has been recently suggested [49,50]. This microorganism was described as motile [45]. The characterization of these motile cells revealed the presence of a single subpolar flagellum (later named as Fla1) (see Figure 2) that promotes a swimming pattern characterized by linear runs interrupted by short stop events. This bacterium swims in liquid medium at average velocities of 80 to 45 μm/s [51,52]. The flagellar motor is dependent on the H^+^ gradient and it rotates unidirectionally interrupted by short stop periods [51,53]. During the stop events the flagellum is locked by an unknown mechanism [54]. The initial characterization revealed that most of the genes encoding for this structure were clustered and its organization in the genome was similar to that found in other well characterized bacteria, such as *Escherichia coli* and *Salmonella enterica* [55]. However, further studies on the molecular structure of this flagellum have shown that it has particular components that evoke those found in *Vibrio* [18,56,57].

When the genome sequence of *R. sphaeroides* was completed, the presence of a second flagellar gene cluster was evident [46]. The cluster was later named *fla2*, given that it could potentially form a complete functional flagellum. However, these genes were apparently not expressed according to microarray studies (data accessible at NCBI GEO database accession, GSE139, and GSE12269) [58,59]. Phylogenetic studies revealed that the *fla2* cluster is vertically inherited in this bacterium, whereas the *fla1* genes were probably acquired by a horizontal transfer event from an ancestral γ-proteobacterium [60]. Later on, we showed that the expression of the *fla*2 genes was possible under specific conditions in the laboratory [61]. Nonetheless, the signals that triggers in nature the expression of these genes remain to be determined. The expression of the second gene cluster gives rise to several polar flagella that, like the Fla1 flagellum, allow *R. sphaeroides* to swim in a liquid medium [60,62] (Figure 2). The number of flagella per cell ranges from two to nine with an average of 4.5 [62]. The chemosensory response of the Fla2 flagella is controlled by a set of CheY proteins, i.e., CheY1, CheY2, CheY5 that, until the *fla*2 cluster was expressed, lacked a motility phenotype when mutated [63,64,65].

The evolution of the Fla1 flagellum has allowed its adaptation to support efficient swimming of this bacterium. In this review we present the outstanding features of this flagellum and the main differences with the *fla2* genetic system.

## 2. Overview of the Flagellar Genetic System in *R. sphaeroides*

The *fla*1 genes are mainly organized in a single locus that also includes several genes related to the chemotactic response of this flagellum, as well as several regulatory genes. This region is located in chromosome I and it is comprised of approximately 56.6 kb; other flagellar genes whose products are part of this flagellum are *motAB*, that are not located within this cluster [46,47,66]. 

The σ^54^ factor (RpoN) together with the RNA polymerase core (E) is responsible of the expression of the *fla1* genes. The gene encoding for this particular sigma factor, *rpoN2*, is located within the *fla1* flagellar locus [67,68]. It should be noted that *R. sphaeroides* has the peculiarity of having four different genes encoding for σ^54^ (*rpoN1* to *rpoN4*), and only σ^54-2^ is responsible for the expression of the flagellar genes [68]. Phylogenetic analysis suggests that the different copies of the *rpoN* genes arose from duplication events followed by selection processes that allowed them to specialize [69]. Eσ^54-2^ also controls the expression of some chemotactic genes as is explained later.

It is known that the σ^54^ factor bound to the catalytic core of the RNA polymerase (E) is unable to form an open complex for transcription initiation. This step requires that an activator protein remodels the DNA-Eσ^54^ complex by hydrolyzing ATP [70,71]. The master activator protein for the expression of the class I flagellar genes is FleQ that together with Eσ^54-2^ promotes the expression of an operon that includes a second σ^54^ activator protein named FleT as well as the genes *fliEFGHIJ*. FleQ forms a heterodimeric complex with FleT and activates the expression of the class III flagellar genes. In this gene class the sigma factor σ^28^, also called FliA, and its anti-sigma protein FlgM are expressed, as well as the genes encoding the components required to complete the basal body, the hook, and the stator proteins MotA and MotB. When the hook is completed, FlgM is exported out of the cell and FliA directs the RNA polymerase to express the class IV flagellar genes such as *fliC* and *fliD* encoding flagellin and the filament cap protein, respectively [55,72] (Figure 2).

In accordance with the expression of the *fla1* genes, FleQ and Eσ^54-2^ also activate the expression of the chemotactic genes located within the flagellar locus, achieving the expression of the cytoplasmic chemotactic receptor *tlpT*, and the chemotactic signal transduction system that includes *cheA4* and *cheA3, cheW4, cheR3, cheB2 and cheY6* [65,73,74]. FliA is responsible for the expression of the chemotactic operon that includes *cheY4* and the chemotactic receptor *mcpG*, which is localized in chromosome II [75]. Other chemotactic components that control rotation of Fla1 are encoded in the chemotactic operon *cheOp*2 that includes *cheY3*, *cheA2*, *cheW2* and *cheW3*, *cheR2*, *cheB1* and *tlpC.* This operon is expressed by the housekeeping σ^70^ factor and also from a promoter dependent on σ^28^ [73,75]. The control of the chemotactic response mediated by these proteins is complex and it has been reviewed elsewhere [74].

On the other hand, the expression of the *fla2* genes requires the absence of the Fla1 flagellum, and the activation of a two-component system, formed by the histidine kinase CckA, the phosphotransferase ChpT and the response regulator CtrA [61]. Details of the mechanisms that control CckA activation and the negative control of Fla1 over Fla2 are currently being studied by our group. Nevertheless, when CtrA is phosphorylated by CckA, the expression of the *fla2* genes is turned on. These genes include those within the *fla2* cluster (of approx. 32 kb), *fliM* and *fliG* that are located elsewhere in chromosome I, as well as *flaA* (flagellin), and its regulators *flaF* and *flbT* that are located in plasmid A [76,77]. Recently it was demonstrated that CtrA also activates the expression of the chemotactic operon *cheOp1* that includes three chemotactic receptors i.e., *mcpA*, *mcpB* and *tlpS*, as well as the chemotaxis genes *cheD*, *cheX*, *cheW1*, *cheR1*, *cheY1*, *cheY2* and *cheY5* [73]. It has also been shown that all these components specifically control the chemosensory response of the Fla2 flagella [64]; CtrA also activates other chemotactic receptors [77]; however, it remains to be tested if these receptors affect the chemotactic response mediated by this flagellum. 

## 3. The Hook and Basal Body

**Figure 3:**
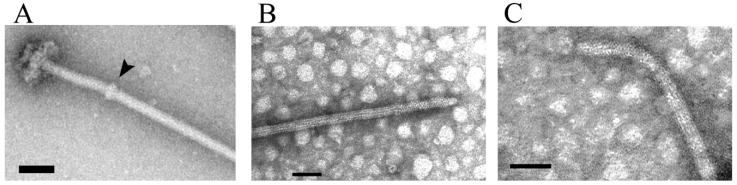
Electron micrographs showing (**A**) wild-type Fla1 filament-hook-basal body (62), arrow denotes the bulky HAP region; (**B**) sheared Fla1 wild-type filament-hook; (**C**) sheared Fla1 filament-hook from a mutant lacking residues 91–96 of FlgE (85). Bar = 50 nm.

Initial characterization of the Fla1 flagellum revealed two prominent features that contrasted from the canonical well-studied flagellum from *E. coli* and *S. enterica.* One of these features was that Fla1 has a straight hook and the second is that it shows a bulky hook-associated-protein (HAP) region [78,79,80] (Figure 3A). The bulky HAP region correlates with the large molecular mass of FlgK1 with 1363 residues, which is three times larger than its homologue in *S. enterica*. FlgK1 has well-conserved N and C-terminal regions with residues present in orthologous proteins, and a large central non-conserved region of 860 residues that accounts for the large molecular mass of this protein. Discrete deletions of 100 amino acids within this non-conserved region revealed that the complete protein is required for normal swimming since practically all these mutants showed a severe reduction in swimming velocity and jiggling trajectories. Importantly, cells expressing FlgK1 lacking residues 340–440 or 840–940 located in the non-conserved region, produced flagella indistinguishable from the wild-type; nevertheless, the mutant cells were unable to swim in liquid medium, revealing that these non-conserved regions are indeed relevant to handle the load exerted by motor rotation [54]. The presence of at least three flagellin-hook IN motifs (pFam07196) detected with the HMMER software package [81] and at least two internal repeats detected with SMART (Simple Modular Architecture Research Tool) [82,83], suggests that this central region could be the result of several processes of internal duplication. So far, few studies have addressed the relevance of the HAP region and its influence on the correct polymorphic shape of the filament when torque is applied [84]. 

As mentioned above, another characteristic feature of the Fla1 flagellum is the presence of a straight hook (Figure 3B). Purified flagella showed a straight hook in a wide range of pH values, from 4 to 9 [66]. This is in contrast with other bacteria such as *E. coli*, *S. enterica*, and the α proteobacterium *Magnetospirillum magnetotacticum* that have a curved hook [66]. The *R. sphaeroides* hook protein FlgE1 is 50% similar to FlgE from *S. enterica* (FlgE_Se_), however it has twice as many proline residues than its counterpart FlgE_Se_ (23/423 versus 12/403), and several of these residues are clustered in short regions not found in FlgE_Se_ [85]. According to the structural model defined for FlgE_Se_ [15,86,87,88], one of these insertions is located in the Dc domain and the other in the D1 domain. A deletion of six residues in one of these regions did not prevent hook assembly but the structure was conspicuously curved (Figure 3C). The swimming trajectories of these cells were wavy instead of the smooth trajectories commonly seen for wild type *R. sphaeroides* cells [85]. This mutation affects the D1 domain that participates in the axial interactions between subunits. Interestingly, it has been recently shown that a short insertion in the Dc domain of FlgE_Se_ made the hook straight. From this study, it was suggested that the Dc domain acts as a structural switch to coordinate axial packing interactions of the D1 domain with the supercoiling of the hook structure [89]. Therefore, these studies concur on the role of the axial packing interactions of D1 domains of the FlgE protein to profoundly affect the final structure of the hook.

## 4. Rod Assembly and Opening of the Peptidoglycan Barrier

Another interesting aspect of the basal body is the order in which the different subunits that form the flagellar rod are assembled. Previously, work in *S. enterica*, showed that FliE and FlgB formed the proximal end of the rod; likewise, previous reports indicated that FlgG is the most distal component. However, the order of assembly was not known i.e., if FlgC or FlgF followed after FlgB. Using purified preparations of the five different rod proteins from *R. sphaeroides*, a possible assembly order was recently suggested. In this study, specific interactions between FliE and FlgB, FlgB and FlgF, and between FlgC and FlgG, were detected. From these results, it was proposed that the order of assembly of the rod proteins in *R. sphaeroides* is FliE, FlgB, FlgF, FlgC and FlgG [90]. This order is different to the one proposed for the Gram-positive bacterium *Bacillus subtilis* and the spirochete *Borrelia burgdorferi*, where it was suggested that the rod proteins are assembled in the following order: FliE, FlgB, FlgC, FlhO (FlgF), and FlgG [40,91]. The difference between the order of assembly proposed for *R. sphaeroides* and *B. subtilis* or *B. burgdorferi* could be explained by the different experimental approaches used in these studies or possibly due to an actual difference between these organisms in the order of assembly of the rod structure. The limited amount of studies that addresses this issue prevent a comparison with species related to *R. sphaeroides*.

Additional proteins are required during the assembly process of this axial structure. In *E. coli* a chaperone protein (FlgJ) has a dual function, as a scaffold and also as a muramidase that degrades the peptidoglycan layer to facilitate rod penetration [9]. In contrast, in *R. sphaeroides* FlgJ lacks the muramidase domain but it retains its ability to function as a scaffold for rod assembly [92]. It was also found that a gene in the *flgG* operon codes for a protein that has a signal sequence at its N-terminus followed by a soluble lytic transglycosylase domain, and could act as a muramidase to remodel the peptidoglycan wall [93]. The protein encoded by this gene is indeed a flagellar soluble lytic transglycosylase named SltF that specifically interacts with FlgJ through its C-terminus. SltF is exported to the periplasm by means of the SecA pathway where it encounters the scaffold protein that directs it to the specific site in the peptidoglycan layer that will be remodeled to allow the passage of the rod [93,94]. Given that SltF is exported by the general secretion pathway, it is possible that this protein must be distributed throughout the periplasmic space potentially causing widespread damage. However, it was recently shown that the enzymatic activity of SltF is modulated by the interaction of the different rod proteins. It is stimulated by the flagellar rod protein FlgB, and inhibited by FlgC, and FlgJ [95]. 

## 5. The Flagellar Motor of *R. sphaeroides*

**Figure 4:**
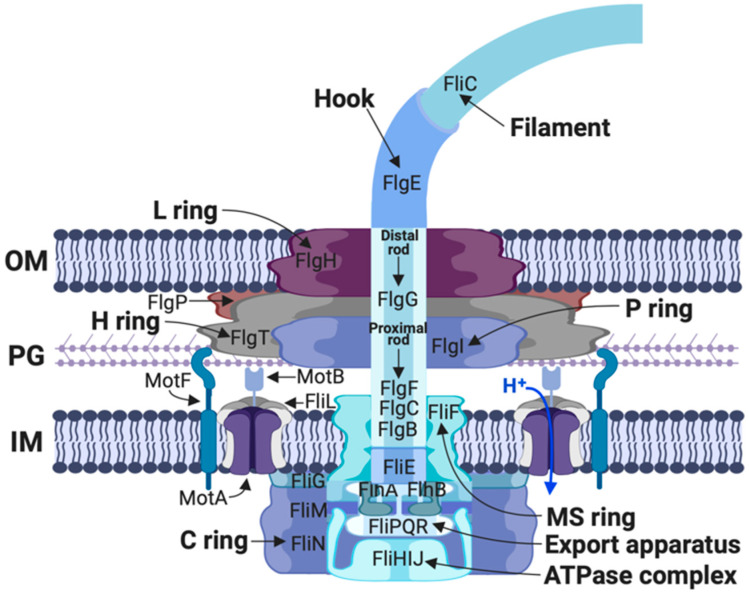
Schematic drawing of the *R. sphaeroides* flagellar motor. The model is based on the electron microscopic analysis of isolated flagella of *R. sphaeroides*, as well as inferences based on protein-protein interaction analysis and in situ visualization of the flagellar structure of *Vibrio*. The name of the different components and proteins that form this structure are indicated. This figure was created with BioRender.com (website: https://biorender.com/).

In the absence of a chemical gradient *R. sphaeroides* swims following a random pattern of runs and stops. During the run periods the Fla1 flagellum rotates unidirectionally in the clockwise direction and uses H^+^ as the coupling ion. When rotation stops, the filament coils up against the cell body, and the swimming trajectory changes [51,96]. Biochemical and genetic studies of the flagellar motor have revealed that, apart from the core structure characterized in *E. coli* and *S. enterica*, other accessory components form part of this flagellum. In this regard it has been shown that in Fla1, proteins homologous to FlgT, FlgP, and MotF (a protein of restricted distribution in some species of the family *Rhodobacteraceae*), are part of this structure (Figure 4).

**Figure 5:**
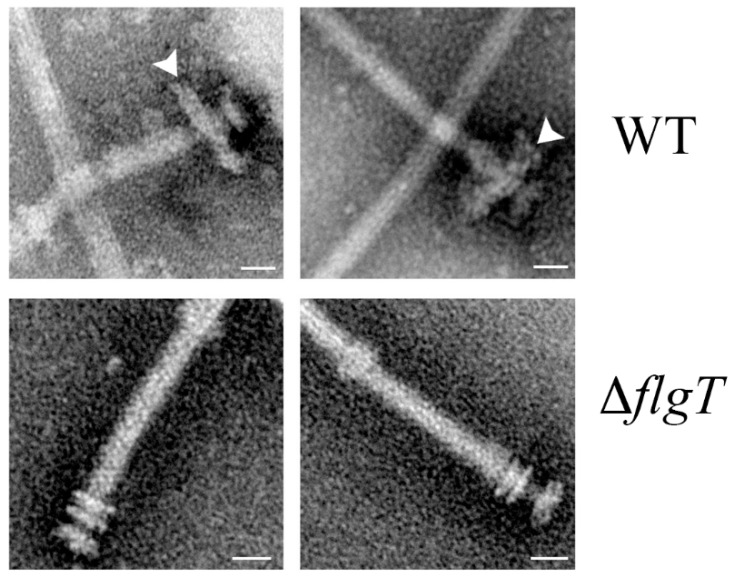
Electron micrographs showing isolated filament-hook-basal bodies from wild type cells expressing Fla1 and from a mutant lacking FlgT. White arrows denote the H-ring Bar = 20 nm [56].

FlgT is a periplasmic protein exported by the general secretion pathway. It forms the H-ring that surrounds the PL-rings and it is widely distributed in several species of *Vibrio*, *Aeromonas*, *Pseudoalteromonas* and also several species of the family *Rhodobacteraceae* [97,98,99]. We have demonstrated that FlgT from *R. sphaeroides* forms a characteristic H-ring (Figure 5), and that this protein, apart from interacting with itself, interacts with FlgH that forms the L-ring of the flagellar core structure, where this interaction would assist to anchor the H-ring to the basal body [56,57]. However, in contrast with the situation observed in *V. alginolyticus*, and *V. cholerae* where the absence of FlgT affects flagellar assembly, as well as the penetration of the outer membrane [97,99,100]; in *R. sphaeroides* the absence of FlgT results in a Mot^−^ phenotype [56]. This indicates that in this bacterium the function of the H-ring is mainly associated with torque generation and motility and not with flagellar assembly. Although its role may not be direct, as discussed below, since FlgT interacts with other proteins that are directly related with torque generation.

The flagellar motor of *R. sphaeroides* also includes the protein FlgP that is an outer membrane lipoprotein essential for flagellum formation [57]. In this work, we observed that FlgP interacts with itself suggesting that it could form an oligomeric structure. It was also proposed that FlgP could form the basal ring that is located under the outer membrane, as it has been previously observed in *C. jejuni* and *V. fisheri* [101]. FlgP also interacts with FlgT and FlgH, these interactions would be an additional support for the formation of the basal disk [57]. Nevertheless, in the absence of FlgT, FlgP should be included given that the flagellar structure is formed; whereas in the absence of FlgP the flagellum is not assembled. More precisely, in the absence of FlgP the flagellar hook is not assembled, even though the hook protein FlgE is present in the cytoplasm. Hence, the anti-sigma factor FlgM is not exported from the cell, and the flagellar genes dependent on σ^28^, such as those encoding for flagellin and other chemotactic proteins, are not expressed. In contrast, in *ΔflgP* mutants the flagellar rod is assembled; therefore, it was proposed that FlgP is required for a proper rod to hook transition [57]. Since, the L-ring assembly has also been related with this process it can be presumed that in *R. sphaeroides* the L-ring could be remodeled by the basal disk. It should be noted that in *V. alginolyticus* it has been proposed that FlgP forms the middle part of the H-ring [100]; however, given the different phenotypes associated with the loss of FlgT and FlgP, we chose to name the structures as H-ring and basal disk respectively, as it was proposed for *V. fischeri* [101]. 

A flagellar gene named *motF* was identified in *R. sphaeroides* and it is present in some species of the family *Rhodobacteraceae*. MotF is a 24 kDa protein that has a transmembrane region spanning from residue 54 to 74 and a large periplasmic C-terminal portion. It was shown that a proper localization of a fluorescent version of this protein is dependent on the presence of an activated proton channel, given that in the absence of MotA/B, or FliL, GFP-MotF forms several fluorescent foci per cell instead of the single one observed in wild-type cells. The presence of several different populations of GFP-MotF in these mutants could be caused by a weak association of GFP-MotF with the flagellar structure when the stator complexes are not present or activated [102].

**Figure 6:**
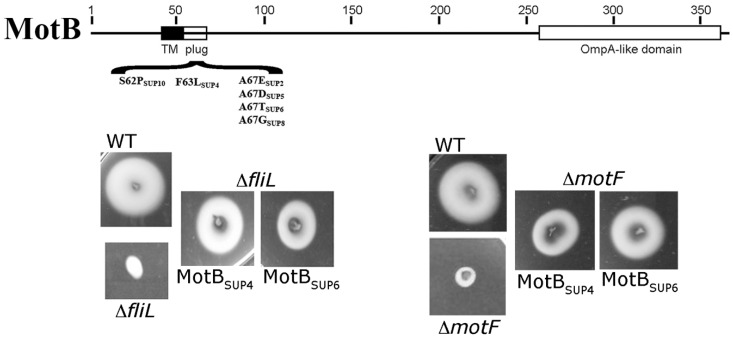
Scheme of the plug region of MotB showing the mutant alleles that suppress the Mot^−^ phenotype of FliL and MotF. Also shown are the swimming phenotypes on soft agar of the various strains and suppressor mutants [34,56,102].

Remarkably, *ΔmotF* cells recovered the swimming ability by a secondary mutation in the amphipathic helix of MotB localized after the transmembrane segment of this protein [102]. This region known as the plug, has been proposed to prevent proton flow before the MotA/MotB complex associates with the flagellar structure [103]. In addition, eight extragenic suppressors of the Mot^−^ phenotype caused by the absence of FliL also affected this specific region of MotB [34]. Surprisingly, all these *motB* mutant alleles were also able to suppress the Mot^−^ phenotype of *ΔmotF* [102] (Figure 6). Therefore, it is strongly suggested that FliL and MotF are implicated in remodeling the C-terminus of MotB and hence promote the activation of the proton channel. If the hydrophobicity of the amphipathic helix of the plug is reduced, as occurs in the suppressor mutants, the presence of FliL and MotF is dispensable for flagellar rotation. 

It was observed that the H-ring (FlgT) is necessary for recruitment of GFP-MotF in the flagellar motor [56]; therefore, the role of FlgT on flagellar rotation could be indirect. In accordance with this possibility we detected that FlgT interacts with FliL and MotF, indicating that the H-ring could act as a hub to recruit or stabilize these proteins that are directly involved in the activation of the proton channel. However, FlgT also interacts with MotB and the mutants in MotB that act as secondary suppressors of *ΔfliL* and *ΔmotF*, barely improve swimming of the *ΔflgT* strain, indicating that the H-ring could also participate in the recruitment of the stator complexes [56]. In this context it is important to mention that in *R. sphaeroides* there are no homologues of *motX* and *motY* whose products have been proposed to form the T-ring in *V. alginolyticus* that contributes to recruit the PomA/PomB (equivalent to MotA/B in *Vibrio*) complexes to the flagellar structure [25,100]. Therefore, FlgT could have possibly evolved in order to gain this role in *R. sphaeroides*.

## 6. Dominance of Fla1 over Fla2

An interesting question regarding the coexistence of the two flagellar systems in this free-living bacterium, is if there is cross-regulation between the two. Under the growth conditions commonly used in the laboratory, Fla2 flagella have never been observed, suggesting that something in the culture medium could favor the expression of the *fla1* genes and repress *fla*2 expression. Routinely, the growth conditions used for the enrichment and isolation of phototrophic bacteria involve the use of organic acids as electron donors in the growth medium, under photoheterotrophic conditions. Therefore, these compounds were obvious candidates to be tested. We have recently demonstrated that the expression of the *cckA* and *ctrA* genes, that encode the histidine kinase and the response regulator of the two-component system that activates the expression of the *fla2* genes, is repressed when a high concentration (34 mm) of C4-dicarboxilic acids is present in the culture medium. However, the growth of the wild-type strain (WS8N) in the absence of C4-dicarboxilic acids is not sufficient to induce activation of the CckA/ChpT/CtrA two-component system and therefore the *fla2* genes are not transcribed. We have speculated that CckA should be activated by additional specific environmental conditions that remain to be understood. Nevertheless, it was observed that the cells carrying a mutation in the master regulator *fleQ* could acquire a gain of function mutation in CckA that allows the expression of the *fla2* genes. As a result, a homogeneous population of bacteria able to swim with the Fla2 flagella was obtained; however, when these cells were complemented with a plasmid expressing FleQ, most of the cells stopped synthesizing Fla2 flagella and the number of cells expressing Fla1 flagella increased sharply. Remarkably, cells carrying both types of flagella were never detected. This suggests that, under laboratory conditions, there is a clear dominance of the Fla1 system over Fla2 mediated by an unknown molecular mechanism [61]. The elucidation of this regulatory mechanism would shed light on how a complete set of foster genes were acquired and stably incorporated into the regulatory circuit that controls motility in this organism and also would reveal details of the evolutionary success of this resourceful bacterium.

## 7. Future Directions

Given that the *fla1* system was laterally acquired, it is important to elucidate the molecular mechanisms controlling biogenesis and rotation of this structure with particular emphasis on FlgP and FlgT, which are not present in the vertically inherited flagellar genes of several α-proteobacteria so far characterized. It is apparent that these proteins do not accomplish the same function that their homologues in *Vibrio*, suggesting that they may have evolved differently. In this context, it would be relevant to look deeper into the molecular role of the family *Rhodobacteraceae*-specific MotF protein and test its possible role as a stabilization element of the stator complexes (MotA/MotB).

Regarding flagellar biogenesis, it will be important to determine the molecular mechanisms underlying the localization and control of the activity of the soluble lytic transglycosylase (SltF) that is important, not only to understand, how a flagellar-specific lytic enzyme is controlled, but also to determine if these mechanisms are conserved in other type III secretion systems. 

The in situ analysis of the structure of the Fla1 motor by combining cryo-EM and genetic studies is an important pending assignment to identify the hypothetic basal ring and the localization of MotF.

The elucidation of the genetic mechanisms that control the communication between the *fla1* and *fla2* genetic systems that results in a mutually exclusive expression, is of particular relevance.
